# Editorial: Tumorigenesis regulated by miRNAs, Volume II

**DOI:** 10.3389/fmolb.2023.1264564

**Published:** 2023-08-09

**Authors:** Taomei Liu, Guangchao Li, Wei Ye

**Affiliations:** ^1^ State Key Laboratory of Applied Microbiology Southern China, Guangdong Provincial Key Laboratory of Microbial Culture Collection and Application, Guangdong Open Laboratory of Applied Microbiology, Guangdong Institute of Microbiology, Guangdong Academy of Sciences, Guangzhou, China; ^2^ Guangzhou Bio-Gene Technology Co., Ltd., Guangzhou, China

**Keywords:** miRNA, tumorgenesis, diagnosis, regulation, treatment

More and more people are suffering from cancer due to the problems of food safety, environmental pollution and increasing pressure. In the year of 2020, near 20 million people are suffering from the cancer all over the world, and this number is increasing continuously. Thus, it is imminent to develop novel strategies to alleviate the hazard of cancer for human being.

Micro RNAs are small non-coding RNAs that participate in the post-transcriptional regulation of gene expression by affecting the stability and translation of transcripts. They are transcribed by RNA polymerase II. The enzyme produces pre-miRNAs blocked by polyadenylation. In addition to tumor-associated proteins and their coding genes, noncoding RNA have been identified as key regulators of many biological processes. MiRNAs have been shown to be involved in the pathogenesis of many diseases, including different types of cancer, arteriosclerosis, ischemic stroke and liver fibrosis with differential expression regulation of miRNAs in various disease mechanisms. Novel high-efficiency methods can be developed to specifically detect cancer by the screening of the most specific mRNA and model diagnosis; new therapeutic drugs could be designed to treat a variety of tumors or neurodegeneration through the regulation of ferroptosis concentration and potentially increasing the effectiveness of these therapies by miRNAs. Therefore, the aim of this study is to develop novel mRNA that can regulate the expression of tumor-associated genes and to develop diagnostic biomarkers for diverse types of tumors. Meanwhile, it is necessary to evaluate the specificity of miRNA-regulated tumors and to develop miRNA-targeted therapies for tumorgenesis.

Previous studies have focused on the regulatory mechanisms of miRNAs in tumor cells, the diagnostic biomarkers of tumors, and the interfering with relevant disease pathology through the emerging role of miRNAs in the regulation of ferroptosis. The four articles in this Research Topic “Volume II: Tumorigenesis regulated by miRNAs” are mainly involved in the following aspects:

1. The molecular mechanism of miRNAs in penicillin-induced persistent pathogen infection and carcinogenesis.
2. The application of circulating mRNA in the diagnosis of breast cancer.
3. miRNA regulates iron concentration to induce the apoptosis of cancer cells.

For instance, the article *Integrating lncRNAs and mRNAs Expression Profiles in Penicillin-Induced Persistent Chlamydial Infection in HeLa Cells* used microarrays to identify lncRNA and messenger RNA that were differentially expressed between *Chlamydia* infection and uninfected cells (Huang et al.). A network of these differentially expressed genes (DEGs) was constructed to elucidate the relationship between lncRNA and mRNA. Gene Ontology (GO) and Kyoto Encyclopedia of Genes and Genomes (KEGG) pathway analysis with differentially expressed genes were also performed. Furthermore, the lncRNA-miRNA-mRNA competing endogenous RNAs (ceRNAs) network. The relative expression levels of four mRNAs were validated by quantitative real-time PCR, the results of which were correlated with the microarray results. In addition, the integration of protein-protein interaction network was constructed and hub genes were identified. These findings provide a new perspective on the molecular mechanisms of penicillin-induced persistent chlamydial infection. The results of LncRNA-miRNA-mRNA networks indicated that the alterations in lncRNA may affect the mRNA transcription and protein translation of vital pathways during the consecutive chlamydialin fection. The findings provide newly discovered information on the critical role of lncRNA in persistent *Chlamydia* infection, which may be useful to know more about the functions of lncRNA. This study showed only an overview of differentially expressed lncRNA and miRNAs by the predication of some tumorgenesis related potential genes and pathways via bioinformatics. Further studies need to be carried out to identify the function of the differentially expressed lncRNAs which are candidates for novel diagnostic biomarkers for chlamydial infection.

The same miRNA shows different regulatory mechanisms in different cancer types. The article *AreviewontheroleofmiR-671in human disorders* illustrates that the MiR-671 gene was involved in the pathogenesis of many diseases (Ghafouri et al.), however, different studies have revealed opposite roles for this miRNA MiR-671. The roles of upregulation and downregulation of MiR-671 under different mechanisms of tumorgenesis were reviewed from three aspects: cytology, animal experiments and human studies. The results in this study indicate that miR-671 is a miRNA that can affect the expression of many target mRNAs in various signaling pathways involved in different human diseases. However, the future application of miR-671 targeted therapy in cancer treatment needs further investigation of *in vitro* and *in vivo* regulatory mechanism of miR-671.

Due to the problem of environmental pollution, food safety and lifestyle, breast cancer (BC) is the most common cancer among women. In 2020, The number of women suffering from breast cancer surpassed the number of lung cancer for the first time, breast cancer has become the leading cause of cancer-related deaths among women worldwide. Nevertheless, the 5-year survival rate for women with early-stage breast cancer is 95 percent. In consequence, early detection of diagnostic biomarkers for BC is one of the most effective ways to reduce thereof mortality. At present, breast cancer is mainly diagnosed through imaging examination, including ultrasound, magnetic resonance imaging and magnetic resonance imaging. There is an urgent need to develop more efficient approaches for specific detection due to the lack of reliable specific biomarkers for the diagnosis of BC. The article *A circulating mir-19b-based model in diagnosis of human breast cancer* collected plasma samples from BC patients and normal controls (Zhao et al.), and determined the differentially expressed miRNAs in the plasma of BC patients. A binary Logit model was used to develop a miRNA diagnostic model, and plasma samples collected from patients with other types of tumors to were used to determine the specificity of this model in BC diagnosis. It was discovered that 5 circulating miRNAs were significantly upregulated in the plasma of BC patients and MiR-19 shows the highest specificity. In combination with miR-16 and miR-106a, a miR-19b-based 3-circulating miRNA model was employed for further validation. Taken the samples together, the model showed 92% of sensitivity and 90% of specificity in the diagnosis of BC. However, further validation using a larger cohort of patients with BC and other tumor types is indispensible for future clinical use of these miRNAs in breast cancer diagnosis.

Ferroptosis is involved in the initiation and progression of many diseases and is regarded as a hotspot of investigations on the treatment of disorders. The article *Emerging role of miRNAs in the regulation of ferroptosis* have shown that microRNAs are involved in regulating the concentration of ferroptosis (Mahmoudi-Lamouki et al.). MiR-675, miR-93, MiR-27a, MiR-34a, and miR-141 have been shown to affect ferroptosis metabolism, antioxidant metabolism, and lipid metabolism, thus affecting all key mechanisms during ferroptosis enrichment. MiRNAs are a small group of non-coding transcripts that specifically bind to their target transcripts and induce their degradation or inhibit their translation. In addition, many miRNAs can affect ferroptosis concentration through indirect pathways. Since ferroptosis enrichment can eradicate cancer cells independently of apoptosis, these miRNAs can induce cancer cell death in different ways. The bioinformatics tool can facilitate the identification of miRNAs involved in ferroptosis concentration and the development of ferroptosis concentration-related miRNA therapies to treat a variety of tumors or neurodegeneration. The combination of miRNA-based therapies with conventional or targeted anticancer therapies is expected to improve the effectiveness of these therapies. Because cancer cells are heterogeneous in terms of miRNA markers, it will be necessary to provide a miRNA profile for each patient before the applications of these new approaches in the clinic.

Previous studies have shown that many miRNAs are found to be associated with cancer, either as tumor suppressors or as oncogenes. Some miRNAs have been discovered to be overexpressed in cancer compared with the corresponding normal tissue, thus suggesting their potential as tumorgenesis related biomarkers. However, the application of these specifically expressed miRNAs in clinical detection and pre-treatment requires more comprehensive screening, alignment, and tumorgenesis regulatory mechanism investigation by cytology, animal experiments, and human experiments. More and more novel targets for cancer cells, such as claudin 18.2, which is a kind of protein responsible for the intercellular molecules communication and highly expressed in the surface of cancer cells due to the destruction of tight junction in cancer cells. Therefore, this new target protein has attracted the attentions of researchers and pharmaceutical companies to develop new anti-cancer drugs such as Zolbetuximab and Osemitamab. Moreover, new miRNAs targeting this kinds of new cancer target proteins can be considered in future study, which needs the extensive screening of the corresponding miRNAs, the *in vivo* and *in vitro* function demonstration and clinical application in diagnosis and treatment, thereby promoting the application of miRNAs in the development of novel anti-tumor drugs.

